# The assessment of health-related quality of life in relation to the body mass index value in the urban population of Belgrade

**DOI:** 10.1186/1477-7525-6-106

**Published:** 2008-11-29

**Authors:** Nadja Vasiljevic, Sonja Ralevic, Jelena Marinkovic, Nikola Kocev, Milos Maksimovic, Gorica Sbutega Milosevic, Jelena Tomic

**Affiliations:** 1 Institute of Hygiene and Medical Ecology Department of Nutrition, Medical School, University of Belgrade, Dr Subotica Street 8, Belgrade 11000, Republic of Serbia; 2 Institute of Medical Statistics and Informatics, Medical School, University of Belgrade, Dr Subotica Street 8, Belgrade 11000, Republic of Serbia

## Abstract

**Background:**

The association between excess body weight, impairment of health and different co-morbidities is well recognized; however, little is known on how excess body weight may affect the quality of life in the general population. Our study investigates the relationship between perceived health-related quality of life (HRQL) and body mass index (BMI) in the urban population of Belgrade.

**Methods:**

The research was conducted during 2005 on a sample of 5,000 subjects, with a response of 63.38%. The study sample was randomly selected and included men and women over 18 years of age, who resided at the same address over a period of 10 years. Data were collected by means of a questionnaire and nutritional status was categorized using the WHO classification. HRQL was measured using the SF-36 generic score. Logistic regression analysis was used to compare HRQL between subjects with normal weight and those with different BMI values; we monitored subject characteristics and potential co-morbidity.

**Results:**

The prevalence of overweight males and females was 46.6% and 22.1%, respectively. The prevalence of obesity was 7.5% in males and 8.5% in females.

All aspects of health, except mental, were impaired in males who were obese. The physical and mental wellbeing of overweight males was not significantly affected; all score values were similar to those in subjects with normal weight.

By contrast, obese and overweight females had lower HRQL in all aspects of physical functioning, as well as in vitality, social functioning and role-emotional.

**Conclusion:**

The results of our study show that, in the urban population of Belgrade, increased BMI has a much greater impact on physical rather than on mental health, irrespective of subject gender; the effects were particularly pronounced in obese individuals.

## Background

In an era of epidemiological and nutritional transition obesity has become an important public health problem, affecting different populations and population groups almost at a pandemic scale [1-4]. Apart from nutrition and lifestyle, health status, social, economic and cultural conditions all contribute to a rise in incidence of obesity in societies where it was hitherto unknown [5-7]. The impact of obesity on national health is also evident in the Serbian population; recent data showed that the prevalence of overweight men and women was 43.0% and 30.9% respectively, whilst the prevalence of obesity was 14.3% in males and 20.1% in females. These results are compatible with the prevalence of obesity recorded in other countries [7]. Problems arising from the increase of obesity in the population include not only the mechanical impact of excess weight and its physical restrictions, the higher morbidity and mortality, but also a significant modification of the general quality of life [8-11]. Whilst obesity has an obvious and often objectively measurable impact on the state of health of the individual, the influence of increased body weight on the subjective perception of the quality of life should not be underestimated [12-14]. Certain adverse effects of excess body weight can be very subtle and it can take some time before they become manifest. In those circumstances self-assessment of the quality of life may suggest that some aspects of physical and mental wellbeing are under threat, thus creating a basis for early recognition and adequate intervention [15,16].

Accordingly, evaluation of the quality of life has become a focus of interest not only in population studies, but also in clinical medicine [17], particularly in patients suffering with chronic illnesses [18-20], where it is assessed with the use of specifically designed instruments. Population research is based on the application of generic instruments such as SF-36, which evaluates both the physical and the mental health of the individual [21]. In studies of obesity the perceived impact of increased body weight on health can be assessed by the use of generic as well as disease-specific assessment scales [22,23]. Population studies evaluating the quality of life have yielded similar results in overweight and obese subjects [24]. However, very few studies are focused on the link between the quality of life and the BMI value in the general population [25,26]. Therefore, the aim of our research was to assess the association between the BMI value and health-related quality of life in the urban population of Belgrade.

## Methods

### Subjects

We carried out a cross-sectional analysis of the quality of life in the urban population of central Belgrade which, according to a recent census, has about 50,000 inhabitants. The study group consisted of a systematic sample (k = 10) of 5,000 subjects over the age of 18 who resided permanently in the area over the last 10 years.

All subjects were handed questionnaires with cover letters which contained information detailing the objectives and methodology of research and consent forms. The participants were then asked to give their written informed consent to join the study, and a timetable was agreed with the interviewers who subsequently collected the completed questionnaires at the appointed time.

### Questionnaires

The survey was anonymous and consisted of two parts. The first part contained questions referring to demographic characteristics of the subject such as sex, age, education, profession, health habits, details of body height and mass, exercise habits, as well as information on possible diseases.

The level of education was divided into four categories which include the following: I – elementary, II – secondary school, III – college and IV – university-level education. A question was asked to ascertain whether the subject was an active smoker.

Physical exercise was divided into two major categories: any form of exercise, excluding walking, lasting a minimum of 30 minutes per day, and walking alone, again for a minimum period of 30 minutes. Both groups where then subdivided according to frequency: 1 – never, 2 – once a month, 3 – once a week, 4 – several times a week and 5 – daily.

The second part of the questionnaire consisted of a short version of the SF-36 generic assessment scale for the quality of life, as an internationally accepted questionnaire on health self-assessment, which has been translated and adapted for the use in Serbian [27].

The SF-36 questionnaire contains 8 scales designed to evaluate physical health as well as mental functioning of the subject. The first four (physical functioning, role-physical, bodily pain and general health) are used to assess physical health whilst the others deal with issues of vitality, social functioning, role-emotional and mental health. The subjects are asked to give answers on a numerical scale; those answers are then coded and assigned a score on a scale of 0–100; a higher score represents a better result in view of the subjective perception of physical and mental health [21].

### BMI categorization

Based on the reported data on body height and mass, BMI values were calculated as the ratio of body mass in kilograms and the square of height in meters. Nourishment status was then evaluated according to the internationally recognized WHO classification: BMI = 18.5–24.9 for normal weight, BMI = 25–29.9 for overweight and BMI >30 kg/m^2 ^for obesity [5]. Underweight subjects were too few in number (n = 16) and were excluded from the study.

### Statistical methods

The subjects were divided into three groups according to nourishment status: normal weight, overweight and obese. For the purpose of sample description, all variables were presented as mean ± SD or frequency, where appropriate. The comparison of results within mean values was carried out by a one-way analysis of variance, with a Bonferroni correction adjusted for multiple comparisons to allow for the three BMI groups. The chi-square test was applied for variables measured on nominal scales.

A stepwise multivariable logistic regression analysis, as a classification tool, was performed to test the association between BMI categories (2 dependent variables: normal vs. overweight, and normal vs. obese) and the 8 scales of SF-36 (the score of each scale as an independent variable) (model 1). Three additional regression models have been tested, adjusting for different sets of covariates: socio-demographic variables, life style variables and health status variables.

Adjustments were made for age and education (model 2) and age, education, smoking, physical exercise other than walking and walking (model 3). Model 4 included the former and major morbidity such as hypertension, diabetes and coronary artery disease. All models, expressed as odds ratios and their 95% confidence interval, were tested separately for men and women. *P *< 0.05 was considered to be statistically significant. All data were recorded and tabulated for analysis using the SPSS 15 for Windows statistical package.

### Ethical approval

The study was reviewed and given ethical approval by the Belgrade Medical School Ethics Committee.

## Results

A total of 5,000 questionnaires were distributed; 3169 were completed and returned (a 63.38% return). Of this number 343 questionnaires were excluded as incomplete so that, ultimately, the sample group consisted of 2,826 subjects who had answered all questions.

The results related to nourishment status are illustrated in Table 1. They revealed a high proportion of overweight males – 46.6%. Normal weight males were significantly younger than overweight and obese males (p = 0.001) and had a higher level of education (p = 0.035). There was, however, no significant difference in smoking habits between normal weight, overweight and obese males. The majority of obese males did not indulge in any form of physical exercise (p = 0.001 for physical exercise other than walking and p = 0.002 for walking).

**Table 1:** Characteristics of the study population, by body mass index (BMI)

	MEN n = 1172				WOMEN n = 1654			
	BMI (kg/m^2^)				BMI (kg/m^2^)			
	18.5–24.9 Normal weight	25–29.9 Overweight	≥ 30 Oobese	*p *Value	18.5–24.9 Normal weight	25–29.9 Overweight	≥ 30 Obese	*p *Value
	
	n = 526 (44.9%)	n = 546 (46.6%)	n = 100 (n = 8.5%)		n = 1168 (70.6%)	n = 365 (22.1%)	n = 121 (7.3%)	

Age(y) (mean ± sd)	39.6 ± 18.9	47.4 ± 17.9	56.8 ± 12.9	0.001	39.2 ± 16.7	54.6 ± 14.4	56.8 ± 12.9	0.001
								
Level of education				0.035				0.001
I	18.0	2.2	12.5		2.2	6.1	12.5	
II	46.0	37.9	45.0		41.7	43.1	45.0	
III	16.8	17.0	13.3		12.3	16.4.	13.3	
IV	35.4	42.9	29.2		43.8	33.4	29.2	
								
Smokers (%)	41.8	37.3	28.2	0.201	44.0	37.4	28.2	0.001
Physical exercise other				0.001				0.001
than walking (%)								
never	30.6	45.9	77.2		50.2	68.8	77.2	
once a month	22.5	22.8	11.9		18.8	17.1	11.9	
once a week	30.2	20.6	5.0		23.0	9.5	5.0	
several times a week	8.5	5.1	0.0		4.3	1.2	0.0	
every day	8.2	5.6	5.9		3.6	3.4	5.9	
Walking (%)				0.002				0.001
never	19.2	28.3	56.4		27.5	40.2	56.4	
once a month	47.7	43.7	37.3		51.5	46.4	37.3	
once a week	21.5	20.9	4.5		16.3	10.8	4.5	
every day	11.5	7.1	1.8		4.7	2.6	1.8	
								
Arterial hypertension (%)	10.9	22.4	53.5	0.001	10.3	36.8	53.5	0.001
Diabetes mellitus (%)	4.3	7.2	11.2	0.001	2.9	8.3	11.2	0.001
Coronary artery disease (%)	5.5	10.1	26.5	0.005	5.4	15.7	26.5	0.001
Myocardial infarction (%)	2.3	3.9	6.4	0.011	1.3	4.4	6.4	0.001
Hypercholesterolemia (%)	8.3	18.4	31.5	0.001	10.7	31.6	31.5	0.001
Hypertrygliceridemia (%)	7.1	18.3	27.5	0.001	6.2	19.6	27.5	0.001

The *p *value is for comparison of means or percentages among men and women using the chi-square test or by ANOVA.

The prevalence of overweight and obese females was 22.1% and 7.5% respectively. There was a significant difference in age between normal weight, overweight and obese women (p = 0.001). Obese woman had a lower level of education than normal weight and overweight women (p = 0.001). There was a significant proportion of smokers among overweight and normal weight by comparison to obese women (p = 0.001). Physical exercise other than walking and walking were practiced less by obese and overweight females when compared to normal weight women (p = 0.001). The incidence of illness increased with higher BMI values; arterial hypertension was most prevalent among obese individuals of both sexes (p = 0.001), followed by hypercholesterolemia (p = 0.001), hypertriglyceridemia (p = 0.001) and coronary artery disease (p = 0.005 for males and p = 0.001 for females).

Comparison of the mean scores of the SF-36 questionnaire on health-related quality of life by body mass index is presented in Table 2. The scores for physical health were the highest reported in normal weight subjects of both sexes. In overweight subjects the highest scores were noted on the role-emotional scale whilst the lowest were obtained on the vitality scale. In obese subjects of both sexes the mean values of physical health scores, with the exception of role-physical, were lower than those of mental wellbeing. Obese males had significantly lower scores for physical functioning (p < 0.001), bodily pain (p < 0.002) and general health (p < 0.003) when compared to men with normal weight.

**Table 2:** Scores of the SF-36 questionnaire on health-related quality of life by body mass index

			BMI(kg/m^2^) men				
	
	18.5–24.9 Normal weight		25.0–29.9 Overweight		≥ 30 Obese		
	
	N = 526		N = 546		N = 100		F
	
	mean(± s.d.)	/rank/	mean(± s.d.)	/rank/	mean(± s.d.)	/rank/	
Physical functioning	90.2 (17.0)**	1	85.0 (19.2)	3	77.1 (22.3) §§	3	23.329
Role-physical	82.1 (30.2)	4	81.1 (30.6)	4	75.2 (32.8)	4	1.959
Bodily pain	79.7 (23.3)*	5	77.6 (23.9)	5	72.9 (25.4) **§**	5	3.629
General health	68.4 (17.3)**	7	66.6 (16.8)	7	62.0 (18.4) **§§**	7	5.660
Vitality	64.7 (18.2)	8	65.6 (17.5)	8	61.5 (19.3)	8	2.120
Social functioning	86.0 (16.5)	2	85.7 (15.8)	2	82.9 (18.3)	1	1.582
Role-emotional	85.0 (30.3)	3	86.5 (28.7)	1	81.6 (34.2)	2	1.144
Mental health	69.7 (17.9)	6	71.0 (15.9)	5	68.6 (17.5)	6	1.181
							
			BMI(kg/m^2^) women				
	
	18.5–24.9 Normal weight		25.0–29.9 Overweight		≥ 30 Obese		
	
	N = 1168		N = 365		N = 121		F
	mean(± s.d.)	/rank/	mean(± s.d.)	/rank/	mean(± s.d.)	/rank/	
Physical functioning	86.6 (20.0)***	1	68.4 (25.6) &&	3	59.4 (28.0) §§	5	130.737
Role-physical	78.3 (32.7)**	4	65.2 (38.5) &&	4	62.4 (36.9) §§	3	25.046
Bodily pain	71.7 (25.6)**	5	62.3 (26.9) &&	6	53.1 (27.9) §§,	7	38.169
General health	66.2 (18.0)**	6	59.5 (18.0) &&	7	54.8 (17.7) §§	6	31.296
Vitality	59.5 20.0)**	8	55.7 (19.7) &&	8	52.1 (22.6) §§	8	9.954
Social functioning	82.6 (17.1)**	3	78.7 (18.0) &&	2	77.1 (20.0) §	2	10.373
Role-emotional	84.2 (31.0)*	2	79.0 (35.0) &	1	79.3 (32.5)	1	4.065
Mental health	65.1 (19.0)	7	64.0 (18.6)	5	62.3 (20.1)	4	1.365

s.d.: standard deviation; F-F statistic, Fischer Anova

*The *P *Value is for overall comparison; & the p value is for comparison between normal weight and overweight; § p value is for comparison between normal weight and obese; by ANOVA.

* p < 0.05;** p < 0.01,*** p < 0.001

§ p < 0.05; §§ p < 0.01;

& p < 0.05; && p < 0.01;

In female subjects all scores for physical and mental health tended to decrease with the rise in BMI values. Scores on all physical health scales differed significantly both in overweight and obese females by comparison to women with normal weight (p < 0.001). Also significant (p < 0.001) were differences between overweight and obese women in scores for physical functioning and bodily pain. The assessment of mental functioning in female participants showed much lower score values in overweight (p < 0.01) and obese subjects (p < 0.01), except on the mental health scale.

Table 3 presents values of the odds ratio of the quality of life scores in relation to nourishment status in men. Physical functioning was considerably lower in overweight men (p < 0.001), whilst other quality of life scores did not differ significantly when compared to normal weight men. In obese males, the probability of lower quality of life in terms of physical functioning (p < 0.001), role-physical (p < 0.05) and bodily pain (p < 0.01) was significantly higher by comparison to normal weight men. Physical functioning was much lower in overweight men (p < 0.05), regardless of age and level of education.

**Table 3:** Odds ratios for 8 domains of SF-36 by BMI categories for men (normal weight vs. overweight, and normal weight vs. obese)

	18.5–24.9	25–29.9		≥ 30	
	Normal weight	Overweight		Obese	

	OR	OR	(95%CI)	OR	(95%CI)

Model 1					
Physical functioning	1	1.96	(1.51–5.54) ***	5.39	(2.97–9.77) ***
Role-physical	1	0.98	(0.75–1.29)	1.64	(1.04–2.58)*
Bodily pain	1	1.15	(0.89–1.48)	1.74	(1.10–2.75)**
General health	1	1.76	(0.63–4.89)	2.12	(0.27–16.73)
Vitality	1	1.73	(0.71–4.23)	1.35	(0.30–6.08)
Social functioning	1	0.99	(0.77–1.27)	1.33	(0.85–2.08)
Role-emotional	1	0.93	(0.69–1.26)	1.28	(0.79–2.10)
Mental health	1	2.15	(0.86–5.37)	2.97	(0.39–22.82)
Model 2					
Physical functioning	1	1.40	(1.04–1.87)*	2.93	(1.53–5.61)
Role-physical	1	0.84	(0.62–1.11)	1.34	(0.82–2.18)
Bodily pain	1	1.03	(0.80–1.34)	1.59	(0.98–2.60)
General health	1	1.27	(0.45–3.62)	0.92	(0.10–8.44)
Vitality	1	1.59	(0.63–4.03)	1.00	(0.19–5.49)
Social functioning	1	0.93	(0.71–1.21)	1.24	(0.77–2.01)
Role-emotional	1	0.85	(0.62–1.16)	1.13	(0.67–1.90)
Mental health	1	1.96	(0.77–5.00)	2.06	(0.25–17.21)
Model 3					
Physical functioning	1	1.42	(0.94–2.16)	3.89	(0.68–22.45)
Role-physical	1	0.81	(0.51–1.27)	1.37	(0.39–4.82)
Bodily pain	1	0.85	(0.57–1.27)	1.55	(0.43–5.54)
General health	1	1.55	(0.33–7.16)	0.96	(0.21–9.65)
Vitality	1	2.72	(0.43–17.2)	0.04	(0.02–1.23)
Social functioning	1	0.92	(0.62–1.36)	1.51	(0.41–5.61)
Role-emotional	1	0.99	(0.62–1.58)	2.01	(0.53–7.66)
Mental health	1	2.89	(0.56–14.86)	1.17	(0.33–4.65)
Model 4					
Physical functioning	1	1.43	(0.91–2.24)	3.36	(0.53–21.37)
Role-physical	1	0.74	(0.46–1.20)	1.16	(0.28–4.76)
Bodily pain	1	0.85	(0.56–1.29)	0.87	(0.22–3.48)
General health	1	1.85	(0.38–9.11)	0.92	(0.36–4.68)
Vitality	1	3.28	(0.39–27.97)	0.22	(0.12-.321)
Social functioning	1	0.99	(0.66–1.51)	0.84	(0.19–3.73)
Role-emotional	1	1.07	(0.65–1.75)	1.87	(0.42–8.22)
Mental health	1	3.13	(0.60–16.21)	1.43	(0.36–7.89)

Model 1 not adjusted; Model 2 adjusted for age and education;

Model 3 adjusted for age, education, smoking, physical exercise other than walking, walking;

Model 4 adjusted for age, education, smoking, physical exercise other than walking, walking, diabetes, hypertension, coronary artery disease

*P *value < 0.001***;*P *value < 0.01**;*P *value < 0.05*

Table 4 shows the odds ratio of quality of life in relation to the nourishment status in women. By comparison to women with normal weight, the overweight group had lower physical functioning (p < 0.001), role-physical (p < 0.001), bodily pain (p < 0.001) and social functioning (p < 0.001) as well as role-emotional (p < 0.01) scores. After adjustment for age, level of education, smoking, physical exercise other than walking, walking, hypertension and coronary artery disease (models 2, 3 and 4), the capacity for physical functioning was still significantly lower in overweight when compared to normal weight females (p < 0.001; p < 0.01; p < 0.05). In obese women, scores for physical functioning (*p *< 0.001), role-physical (*p *< 0.001), bodily pain (*p *< 0.001), social functioning (*p *< 0.05) and role-emotional (*p *< 0.01) were much lower in comparison to normal weight woman. After adjustment, as outlined above for models 2, 3 and 4, the odds ratio differences were eliminated.

**Table 4:** Odds ratios for 8 domains of SF-36 by BMI categories for women (normal weight vs. overweight, and normal weight vs. obese)

	18.5–24.9	25–29.9		≥ 30	
	Normal weight	Overweight		Obese	

	OR	OR	(95%CI)	OR	(95%CI)

Model 1					
Physical functioning	1	5.98	(4.01–8.93) ***	12.04	(4.91–29.98) ***
Role-physical	1	1.94	(1,51–2.49) ***	2.64	(1.76 – 3.97)***
Bodily pain	1	1.72	(1.30–2.99) ***	2.56	(1.52–4.30)***
General health	1	2.22	(0.66–7.44)	2.34	(0.31–17.47)
Vitality	1	2.23	(0.51–9.80)	0.77	(0.17–3.40)
Social functioning	1	1.57	(1.18–2.07) ***	1.53	(0.99–2.38) *
Role-emotional	1	1.39	(1.06–181) **	1.62	(1.08–2.42) **
Mental health	1	1.29	(0.37–4.57)	1.31	(0.17–10.14)
					
Model 2					
Physical functioning	1	2.87	(1.87–4.40)***	1.34	(0.79–2.27)
Role-physical	1	1.27	(0.96–1.68)	1.14	(0.74–1.73)
Bodily pain	1	1.21	(0.89–1.64)	1.00	(0.65–1.55)
General health	1	0.84	(2.22–3.14)	2.31	(0.31–17.56)
Vitality	1	2.32	(0.49–10.91)	1.24	(0.39–2.16)
Social functioning	1	1.28	(0.95–1.74)	0.83	(0.54 – 1.27)
Role-emotional	1	1.12	(0.84–1.51)	1.13	(0.72–1.75)
Mental health	1	1.57	(0.41–6.02)	1.51	(0.19–11.78)
					
Model 3					
Physical functioning	1	2.64	(1.27–5.4) **	1.17	(0.44–3.12)
Role-physical	1	1.05	(0.55–2.01)	0.67	(0.26–1.76)
Bodily pain	1	1.17	(0.61–2.24)	0.98	(0.41–2.38)
General health	1	0.23	(0.59–2.55)	0.85	(0.94–3.42)
Vitality	1	1.25	(0.98–3.26)	0.96	(0.64–2.68)
Social functioning	1	0.95	(0.50–1.80)	0.48	(0.20–1.14)
Role-emotional	1	0.71	(0.34–1.51)	1.48	(0.59–3.69)
Mental health	1	0.43	(0.39–6.87)	1.39	(0.78–2.66)
					
Model 4					
Physical functioning	1	2.21	(1.0–4.93) *	1.19	(0.44–3.19)
Role-physical	1	1.23	(0.60–2.49)	0.64	(0.24–1.71)
Bodily pain	1	1.43	(0.69–2.98)	0.91	(0.37–2.24)
General health	1	0.28	(0.03–2.86)	0.68	(0.44–2.13)
Vitality	1	0.35	(0.24–1.63)	0.47	(0.38–1.28)
Social functioning	1	1.18	(0.58–2.38)	0.45	(0.19–3.73)
Role-emotional	1	0.84	(0.38–1.89)	1.60	(0.63–4.04)
Mental health	1	0.32	(0.29–1.26)	0.46	(0.28–2.11)

Model 1 not adjusted; Model 2 adjusted for age and education;

Model 3 adjusted for age, education, smoking, physical exercise other than walking, walking;

Model 4 adjusted for age, education, smoking, physical exercise other than walking, walking, diabetes, hypertension, coronary arterial disease

*P *value < 0.001***;*P *value < 0.01**;*P *value < 0.05*

## Discussion

Our study is the first to address the link between perceived HRQL and self-reported weight status in the Serbian urban population, and the first to use the SF-36 generic assessment scale outside the clinical setting [28,29]. We would like to highlight the importance of the application of SF-36 in a society undergoing transition, not only in economic but also in terms of epidemiology and nutrition.

The prevalence of overweight and obese individuals in our sample is compatible with general data on the Serbian population contained in the latest WHO report [7] and with the results of Serbia's National Health Survey of 2006 [30]. According to those, overweight is the dominant category for adult males, while obesity is equally distributed between males and females. The prevalence of overweight and obesity in the population studied corresponds to the results from other European countries and the USA [7,31]. Although our analysis was based on reported rather than measured statistics, as discussed previously, our results were, nevertheless, in keeping with those based on measured data [12,18]. Likewise, other research focused on quality of life, based on self-reported data, yielded results compatible with ours [32-35], as did studies using measured data [25,26] or other instruments to assess HRQL [36].

Our conclusions that increased values of BMI affect the quality of life, and particularly the physical health of the individual, coincide with those of other authors who also found that overweight and obesity have a greater impact on physical rather than mental health [22,25,31,34,35,37-40]. Similarly, we confirmed the findings of other researchers regarding gender differences; both physical and mental health rated higher on all scales in male than in female participants [25,34,35,41].

Overweight, as a nutritional status, appears to have little impact on the subjective perception of physical health in male subjects, except in the realm of physical functioning, a finding also borne out by other authors [36,41-45]. However, assessment of quality of life in the female population shows lower scores in the realm of both physical and mental health in overweight by comparison to normal weight women; this, again, is in agreement with the findings of other studies [18,30,34,44,46-48].

We confirmed the association between obesity and lower HRQL in male participants, highlighted by other authors [25,34-36,44,45,49-51]. In female subjects higher BMI values are associated with lower scores for physical health, as borne out by other research [18,30,34,46,47,52].

Age is also an important determinant of the quality of life, both from the standpoint of physical health and that of mental wellbeing. In our study, overweight female subjects were older than overweight males, who were usually younger and had a better quality of life on all scales, as shown in other studies [34,35,53,54]. In addition, we confirmed the association between overweight and obesity and numerous co-morbidities, which also diminish the quality of life; the morbogenic influence of increased body weight was more dominant in females [18,33,36,55-57]. Our participants had lower levels of physical activity in leisure time, also demonstrated by other authors [58-62].

Increased BMI values have a lesser influence on mental health, which may indicate that vitality, emotional changes, social isolation and mental health impairment are a consequence, rather than a cause of the increase in body mass [32,34,35]. Vitality showed the lowest scores, as confirmed by other studies [48,52,54]. However, extremely high BMI values can have a considerable impact on mental health (fat phobia) [25,48,62], particularly in the female population, which appears more sensitive to stressful situations associated with the modern way of life [25,31,34,62].

The results of our study were confirmed by a logistic regression model which linked high BMI values to lower quality of life. The effect of overweight was particularly prominent in the realm of physical functioning, which was confirmed in both sexes after adjustments for age and education (Model 2). However, after adjustments for age, education, smoking, physical exercise other than walking and walking (Model 3), the lower HRQL was independently linked to overweight only in females. Similar results were obtained in model 4 which also included adjustments for diabetes, hypertension and coronary artery disease (Model 4). Adjustments for exercise behaviour or leisure time physical activity were used by other authors [35,41,63]; we also added walking as a separate form of physical exercise, not included in other research [31,34,41].

The link between obesity and lower physical functioning, role-physical and bodily pain scores was demonstrated in both sexes; in addition, obese female participants had lower social functioning and role-emotional scores. However, after adjustment for other variables (models 2, 3, and 4) no association persisted between obesity and HRQL.

There appeared to be no independent link between various aspects of physical and mental health and the high BMI values in obese individuals, with the exception of physical functioning, which remained related to BMI in both sexes after adjustment for age and education (model 2). However, in male participants this association disappeared after adjustment for lifestyle variables (model 3). The results, therefore, indicate that socio-demographic and lifestyle variables play a more important role in determination of HQRL scores than BMI value, which could be regarded as an intermediate variable.

Although our results are compatible with those of similar research in other population groups/other countries, there are some limitations inherent in our methodology. Firstly, our study was cross-sectional; there was no follow-up of participants to show whether changes in body weight and health behaviour brought about a change of the perceived quality of life. In addition, we used a generic instrument to measure HRQL, not an obesity-specific questionnaire. Hence, we feel that it would be extremely useful to analyze the quality of life on a sample of obese subjects undergoing obesity treatment, compared to a general population sample, to include measurements of body weight and height, using both an SF-36 questionnaire and a specific Impact of Weight on Quality of Life scale. Our aim would be to determine the differences in the quality of life of the overweight and obese, with and without co-morbidities, and reassess and compare the self-reported and measured data. Such an analysis would probably make it possible to ascertain the subtle differences which contribute to a change in the perceived quality of life in individuals with increased body weight, and particularly in the obese.

## Conclusion

The SF-36 questionnaire can be used to in the assessment of physical and mental health in relation to perceived body weight in the urban population of Belgrade. The results of our study confirm that BMI values are associated with the quality of life in both males and females. Results of this type of research, conducted on population samples in diverse natural, social, economic and cultural environments, should be compared to identify the factors leading to increased body weight and obesity and, consequently, to the impairment of health-related quality of life.

## Competing interests

The authors declare that they have no competing interests.

## Authors' contributions

NV did the study concept and design. SR participated in data integration and data analysis accuracy. JM and NK were responsible for statistical analysis and data presentation. MM completed the interpretation of data. GSM carried out a critical revision of the manuscript for important intellectual content. JT was involved in administrative and technical support. All authors had full access to all data, read the manuscript and approved the final version.
